# HLA Class II Genotype Does Not Affect the Myelin Responsiveness of Multiple Sclerosis Patients

**DOI:** 10.3390/cells9122703

**Published:** 2020-12-17

**Authors:** Judith Derdelinckx, Irene Nkansah, Naomi Ooms, Laura Van Bruggen, Marie-Paule Emonds, Liesbeth Daniëls, Tatjana Reynders, Barbara Willekens, Patrick Cras, Zwi N. Berneman, Nathalie Cools

**Affiliations:** 1 Laboratory of Experimental Hematology, Vaccine and Infectious Disease Institute (VaxInfectio), Faculty of Medicine and Health Sciences, University of Antwerp, 2610 Antwerp, Belgium; irene.nana.nkansah@gmail.com (I.N.); Naomi.Ooms@uza.be (N.O.); Laura.VanBruggen@uantwerpen.be (L.V.B.); barbara.willekens@uza.be (B.W.); Zwi.Berneman@uza.be (Z.N.B.); nathalie.cools@uza.be (N.C.); 2 Division of Neurology, Antwerp University Hospital, 2650 Edegem, Belgium; tatjana.reynders@uza.be (T.R.); patrick.cras@uza.be (P.C.); 3 Histocompatibility and Immunogenetics Laboratory, Red Cross-Flanders, 2650 Mechelen, Belgium; Marie-Paule.Emonds@rodekruis.be (M.-P.E.); Liesbeth.Daniels@rodekruis.be (L.D.); 4 Born Bunge Institute, Translational Neurosciences, Faculty of Medicine and Health Sciences, University of Antwerp, 2610 Antwerp, Belgium; 5 Center for Cell Therapy and Regenerative Medicine, Antwerp University Hospital, 2650 Edegem, Belgium

**Keywords:** multiple sclerosis, myelin responsiveness, HLA class II genotype, antigen-specific treatment

## Abstract

Background: When aiming to restore myelin tolerance using antigen-specific treatment approaches in MS, the wide variety of myelin-derived antigens towards which immune responses are targeted in multiple sclerosis (MS) patients needs to be taken into account. Uncertainty remains as to whether the myelin reactivity pattern of a specific MS patient can be predicted based upon the human leukocyte antigen (HLA) class II haplotype of the patient. Methods: In this study, we analyzed the reactivity towards myelin oligodendrocyte glycoprotein (MOG), myelin basic protein (MBP) and proteolipid protein (PLP) peptides using direct interferon (IFN)-γ enzyme-linked immune absorbent spot (ELISPOT). Next, the HLA class II haplotype profile was determined by next-generation sequencing. In doing so, we aimed to evaluate the possible association between the precursor frequency of myelin-reactive T cells and the HLA haplotype. Results: Reactivity towards any of the analyzed peptides could be demonstrated in 65.0% (13/20) of MS patients and in 60.0% (6/10) of healthy controls. At least one of the MS risk alleles HLA-DRB1*15:01, HLA-DQA1*01:02 and HLA-DQB1*06:02 was found in 70.0% (14/20) of patients and in 20.0% (2/10) of healthy controls. No difference in the presence of a myelin-specific response, nor in the frequency of myelin peptide-reactive precursor cells could be detected among carriers and non-carriers of these risk alleles. Conclusion: No association between HLA haplotype and myelin reactivity profile was present in our study population. This complicates the development of antigen-specific treatment approaches and implies the need for multi-epitope targeting in an HLA-unrestricted manner to fully address the wide variation in myelin responses and HLA profiles in a heterogeneous group of MS patients.

## 1. Introduction

In autoimmune diseases, immune responses are derailed causing immunity towards self-proteins. In the particular case of multiple sclerosis (MS), loss-of-tolerance towards myelin proteins is presumed to underlie the immunopathogenesis of the disease. Although the exact cause for this breach in tolerance to myelin-derived structures has not yet been fully deciphered, T cell responses towards various myelin proteins, including myelin basic protein (MBP), myelin oligodendrocyte glycoprotein (MOG), proteolipid protein (PLP) and alpha B-crystallin, have been demonstrated in MS patients [1].

Although patient-tailored treatments would benefit from prior knowledge of the targeted myelin-derived epitopes, no single auto-antigen is being targeted in MS, nor does a specific pattern of myelin reactivity exist. On the contrary, myelin reactivity has been demonstrated to be patient-dependent [2,3] as well as time-dependent, as indicated by fluctuations in the myelin reactivity profile occurring over the course of the disease. Both disappearance and re-emergence of reactivity towards specific peptides [2], as well as expansion of the reactivity profile to additional myelin-derived peptides [4,5,6,7] have been described. The latter is known as epitope spreading and is believed to play an important role in the progression of the disease, as observed both in MS and in its animal model, experimental autoimmune encephalomyelitis (EAE) [5,6,8]. Indeed, a consistent cascade of epitope spreading was demonstrated during the progression of EAE in (SWR × SJL)F1 mice immunized with PLP_139–151_ [9]. Both intramolecular spreading to other epitopes of PLP and intermolecular spreading towards MBP was demonstrated, accompanied by clinical progression of the disease [9]. In our hands, intra- and intermolecular epitope spreading could be detected to the same extent in a MOG_35–55_-induced C57BL/6 EAE mouse model [10]. Similarly, a decline in primary autoreactivity was followed by emergence of reactivity towards new epitopes in MS patients, although the specific pattern of epitope spreading was highly patient-dependent [5,6]. Better insights in human myelin-specific reactivity profiles could contribute to the development of next-generation antigen-specific therapies adapted to the patients’ needs.

T cells require three signals for full antigen-specific stimulation, i.e., (i) interaction of the T cell receptor (TCR) with antigen peptide bound to human leukocyte antigen (HLA)-molecules on the surface of antigen-presenting cells (APC), (ii) triggering of T cell-bound CD28 by APC-bound costimulatory molecules CD80 and CD86 and (iii) the presence of polarizing cytokines [11]. There is evidence that cytokines secreted by activated CD4+ T helper 1 (Th1) and Th17 cells, including interferon (IFN)-γ, interleukin (IL)-17 and tumor necrosis factor (TNF)-α, play a critical role in the immune pathogenesis of MS and its animal model EAE [12]. Hence, not surprisingly, the best-described heritable risk factors for MS lie within the class II region of the HLA gene cluster [13], involved in the presentation of various antigens—including myelin antigens—to these CD4+ T lymphocytes. Three main HLA class II molecules participate in the presentation of antigen peptides to CD4+ T cells, i.e., HLA-DR, -DP and -DQ. The strongest association with MS has been demonstrated for HLA-DRB1*15:01, which is found in higher frequency among MS patients as compared to healthy controls and which is associated with an increased risk of MS development among carriers of one or both alleles (average odds ratio 3.08; [14]). The HLA-DRB1*15:01 allele is often combined with HLA-DQB1*06:02 and HLA-DQA1*01:02 in the HLA-DR15 haplotype [15]. Additional MS risk alleles include HLA-DRB1*13:03, HLA-DRB1*03:01, HLA-DRB1*08:01, HLA-DQB1*03:02 [16] and HLA-DPB1*03:01 [17].

Promiscuous or degenerate binding to HLA-DR molecules has been demonstrated for several peptides [18,19,20], including myelin-derived peptides [21,22]. This implies the presence of binding affinity to multiple HLA-DR molecules for these peptides. Both MBP peptides, including MBP_13–32_, MBP_84–102_, MBP_84–103_ and MBP_144–163_ [21,22], and MOG peptides, including MOG_146–154_ [23], have been shown to display such degenerate binding. Interestingly, one of the peptides displaying the most degenerate binding, MBP_84–103_, was demonstrated to bind to both MS-associated and non-MS-associated HLA susceptibility molecules, albeit with lower affinity to the latter [21]. On the other hand, some MBP peptides displayed a more restricted binding to one or two HLA-DR molecules [21]. Therefore, it remains elusive whether an association between the myelin reactivity pattern and the HLA class II haplotype exists. Nonetheless, predicting the myelin reactivity profile of a particular MS patient based on the HLA haplotype would facilitate patient selection and choice of antigens for antigen-specific tolerance-inducing treatment strategies.

In this study, we analyzed the reactivity towards MOG, MBP and PLP peptides and compared this with the HLA class II haplotype profile. In doing so, we aimed (1) to characterize the myelin reactivity and HLA class II profile in a Belgian population of MS patients and healthy controls and (2) to evaluate the possible association between precursor frequency of myelin-reactive T cells and HLA haplotype.

## 2. Materials and Methods

### 2.1. Study Population and Ethics

Ten healthy volunteers and 20 relapsing-remitting MS (RR-MS) patients, diagnosed according to the 2010 McDonald criteria [24], were included. Patients were untreated or treated with first-line disease-modifying treatments, i.e., interferons, glatiramer acetate, teriflunomide or dimethyl fumarate. Approximately 100 mL of blood was collected by venous puncture and was further processed for myelin reactivity screening or HLA class II haplotyping. All subjects gave written consent for participation in the study. The study was approved by the Ethics Committee of the Antwerp University Hospital and the University of Antwerp (study number 16/11/138) and followed the Tenets of Helsinki.

### 2.2. Myelin Reactivity Screening

Peripheral blood mononuclear cells (PBMC) were isolated by density gradient centrifugation (Ficoll-Plaque PLUS, GE Healthcare, Chalfont St. Giles, UK). Subsequently, PBMC were resuspended in Iscove’s Modified Dulbecco’s Medium (IMDM) supplemented with 5% human AB serum (hAB) at a concentration of 4 × 10^6^ cells/mL and were prestimulated with a peptide mix containing the following myelin peptides: MOG_1–20_, MOG_35–55_ (both Hemmo Pharmaceuticals Private Limited, Mumbai, India), MOG_64–86_, MOG_74–96_ (both Severn Biotech Ltd., Kidderminster, UK), MBP_13–32_, MBP_34–56_, MBP_111–129_, MBP_146–170_ (all Hemmo Pharmaceuticals), MBP_30–44_, MBP_131–145_ (both Pepscan, Lelystad, the Netherlands) and PLP_139–154_ (Hemmo Pharmaceuticals), at a concentration of 10 nM for each peptide. After 6 days, PBMC were tested for myelin reactivity using direct interferon (IFN)-γ enzyme-linked immune absorbent spot (ELISPOT) (Mabtech, NackaStrand, Sweden), according to the manufacturer’s instructions. For this, 2 × 10^5^ cells were restimulated per well with each peptide individually (10 nM) for 18 h at 37 °C in a 96-well plate (Millipore, Bedford, MA, USA). Experiments were performed in triplicate. Unstimulated PBMC served as a negative control. PBMC stimulated with 1 µg/mL phytohemagglutinin (PHA; Sigma-Aldrich, Bornem, Belgium) were used as a positive control. Frequencies of IFN-γ-secreting cells were calculated based on the number of spots counted using an automated AID ELISPOT Reader system (AID GmbH, Strassberg, Germany) and analyzed using AID ELISPOT software version 5.0. A peptide-specific response was defined as present when >50% of the peptide-restimulated wells were positive. A well was defined as positive when the number of spot-forming cells in the well was higher than [mean spot forming cells + 3 standard deviations (S.D.)] of the negative control wells. A subject displaying at least one peptide-specific response was defined as a responder.

### 2.3. HLA Class II Haplotyping

HLA typing was performed for the following loci: HLA-DRB1, -DRB3, -DRB4, -DRB5, -DQA1, -DQB1, -DPA1 and -DPB1. For this, samples were tested with an IMMUCOR^®^ MIA FORA NGS HLA Typing kit (Immucor, Georgia, USA) on the Illumina MiSeq platform (Illumina, Inc., San Diego, CA, USA) and analyzed with MIA FORA IMMUCOR^®^ MIA FORA NGS HLA Typing kit software v3.1.

### 2.4. Data Analysis

Data were analyzed using Graphpad Prism software version 5.01 (Graphpad, San Diego, CA, USA). For comparisons, Fisher’s exact test, unpaired students *t*-test, two-way analysis of variance (ANOVA) and Pearson’ correlation test were used when appropriate. Any *p*-value < 0.05 was considered as statistically significant.

## 3. Results

### 3.1. Study Subjects’ Characteristics

Twenty relapsing-remitting MS patients (12 female, 8 male) with a median age of 44 years (range: 24–69 years) and a median Expanded Disability Status Scale (EDSS) score of 2.0 (range: 0–6.5) were included (Table 1). Two patients had active MS, as defined by the presence of either clinical or radiological disease progression during the last 12 months. Fourteen patients were treated with first-line disease-modifying treatments, while six patients did not receive any treatment. Ten age- and gender-matched healthy controls (6 female, 4 male) with a median age of 42.5 years (range: 22–64) were included.

### 3.2. The Myelin Reactivity Profile of MS Patients and Healthy Controls Displays a High Inter- and Intramolecular Variability

**Figure 1:**
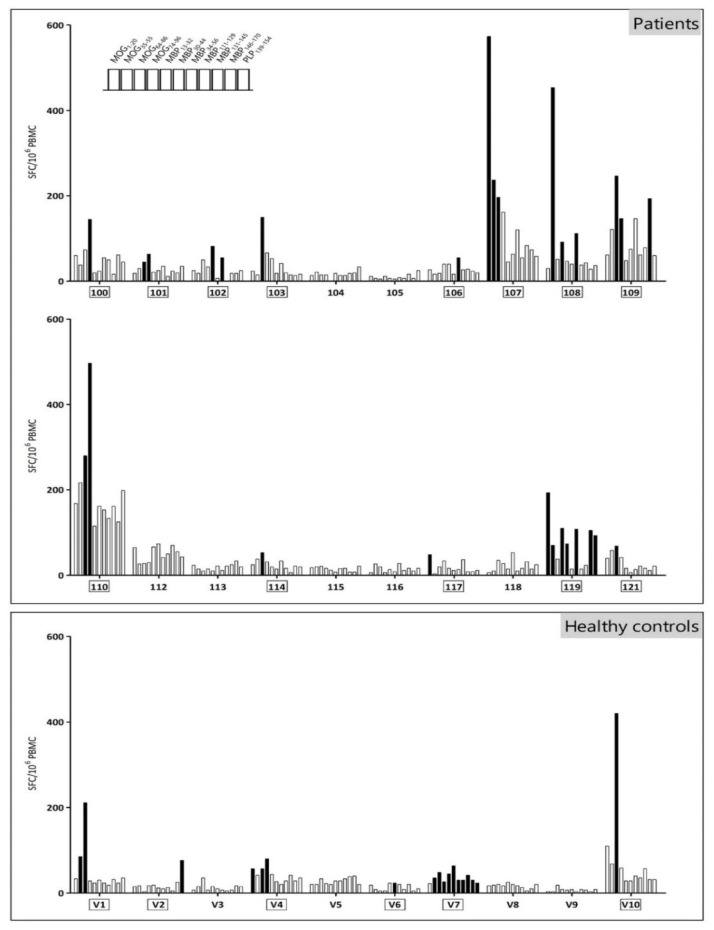
Myelin reactivity screening in 10 healthy controls and 20 MS patients, untreated or treated with first-line immunomodulatory therapy. Per patient or healthy control, each bar represents the IFN-γ response towards the different peptides in the following order: MOG_1–20_-MOG_35–55_-MOG_64–86_-MOG_74–96_-MBP_13–32_-MBP_34–56_-MBP_111–129_-MBP_146–170_-MBP_30–44_-MBP_131–145_-PLP_139–154_. Black bars indicate a positive peptide response, defined as present when >50% of the peptide-restimulated wells were positive. A well was defined as positive when the number of spot-forming cells in the well was higher than the [mean spot forming cells + 3 standard deviations (S.D.)] of the negative control wells. Responder subjects are denoted by a box around the subject code; a responder was defined as a subject demonstrating at least one positive myelin peptide response. Abbreviations used: SFC, spot forming cells; PBMC, peripheral blood mononuclear cells; MOG, myelin oligodendrocyte glycoprotein; MBP, myelin basic protein; PLP, proteolipid protein.

Using IFN-γ ELISPOT, we analyzed the myelin reactivity pattern for MOG, MBP and PLP. Thirteen out of 20 patients (65.0%) and 6 out of 10 healthy controls (60.0%) showed reactivity to at least one of the myelin-derived peptides and were defined as myelin-specific responder (Figure 1 and Table 2). High inter- and intramolecular variability of myelin-specific responses were observed. The majority of myelin-specific responses was directed towards MOG epitopes both in MS patients (19 out of 28 positive responses) and in healthy volunteers (9 out of 18 positive responses), with MOG_64–86_ being the peptide recognized most in all study subjects. MBP-responders were scarcer (5 out of 20 patients and 2 out of 10 healthy controls), and only one PLP_139–154_ responder could be detected among the MS patients. No significant difference in the number of responses between MS patients and healthy controls could be observed (mean number of responses among MS patients 1.4; among healthy controls 1.8; *p* = 0.87), neither did the number of subjects responding to multiple myelin peptides differ between MS patients (7 out of 13 patients; 53.8%) and healthy controls (3 out of 6 healthy controls; 50.0%). Additionally, large variations in the frequency of IFN-γ-secreting myelin-reactive T cells were observed, ranging from 3 to 573 spot-forming cells/10^6^ PBMC (Figure 1). Neither the number of myelin-specific responses nor the frequency of myelin-reactive T cells were influenced by disease duration (R^2^ 0.00005, *p* = 0.98 for number of myelin-specific responses; R^2^ varying between 0.004 to 0.06 among the 11 peptides analyzed; all *p* > 0.05) or EDSS score (R^2^ 0.003; *p* = 0.81 for number of myelin-specific responses; R^2^ varying between 0.0002 to 0.05 among the 11 peptides analyzed; all *p* > 0.05). No effect of gender could be demonstrated for the presence of any myelin response, nor on the total number of myelin responses.

### 3.3. The Majority of MS Patients Carries MS-Associated HLA Risk Alleles

Next, we analyzed the HLA class II genotype of the included subjects, focusing on HLA-DRB1, -DRB3, -DRB4, -DRB5, -DQA1, -DQB1, -DPA1 and -DPB1 (Table 3). Eight out of 20 patients (40.0%) carried the HLA-DR15 haplotype, consisting of HLA-DRB1*15:01, HLA-DQA1*01:02 and HLA-DQB1*06:02. In contrast, only 1 out of 10 healthy controls (10.0%; *p* = 0.20) carried the HLA-DR15 haplotype. This resulted in an odds ratio for HLA-DR15 carriers of 6.00 (95%-confidence interval [0.6315–57.0059]; *p* = 0.11). Moreover, 14 out of 20 patients (70.0%) and 2 out of 10 healthy controls (20.0%; *p* = 0.02) carried at least one of the MS-associated alleles.

### 3.4. The HLA Class II Genotype Does Not Affect Myelin-Specific Responsiveness

Since we demonstrated no differences between the myelin-specific reactivity profile or HLA class II genotype among MS patients and healthy controls, results from both subject groups were pooled to analyze the possible association between the myelin-specific reactivity profile and HLA class II genotype in our study population. Both carriers and non-carriers of the primordial MS-associated HLA risk alleles, i.e., HLA-DRB1*15:01, HLA-DQA1*01:02 and HLA-DQB1*06:02—either alone or combined in the HLA-DR15 haplotype—demonstrated myelin responsiveness to at least one of the tested peptides in the same extent (Table 4). Additionally, no differences in the presence of a myelin-specific response could be detected between carriers and non-carriers of the HLA-DRB3 allele (14 responders out of 22 carriers versus 5 responders out of 8 non-carriers; *p* = 1.0), the HLA-DRB4 allele (5 responders out of 9 carriers versus 14 responders out of 21 non-carriers; *p* = 1.0) or the HLA-DPB1*03:01 allele (2 responders out of 3 carriers versus 17 responders out of 27 non-carriers; *p* = 1.0). Equally, no difference in the number of responders to the individual peptides, nor in the frequency of myelin peptide-reactive precursor cells, could be detected between carriers and non-carriers of HLA-DRB1*15:01, HLA-DQA1*01:02 and HLA-DQB1*06:02, individually or combined as the HLA-DR15 haplotype (Table 4).

## 4. Discussion

Identifying patients that would benefit from treatment with a particular therapy may aid in future patient-tailored therapeutic management of the disease. Especially for antigen-specific therapies, predicting the myelin-specific reactivity profile would allow patient stratification depending on the risk for epitope spreading and choice of antigens for tolerance-inducing treatment strategies. In this context, HLA haplotype may be an interesting parameter for patient stratification, given that some myelin peptides are HLA-restricted [11,12], meaning that they are preferentially presented by specific HLA-molecules. Thus far, different studies investigated the myelin reactivity of a selected group of HLA-DR15-positive patients [25,26,27]. However, no association between the myelin reactivity of carriers and non-carriers of this risk haplotype could be made. Nonetheless, only a few myelin peptides have been demonstrated to be restricted to HLA-DR15, including MBP_84–102_ [27,28], whereas other myelin peptides, including MBP_149–170_ [28], show no specific HLA-DR15 restriction. Hence, additional HLA haplotypes may be of interest for the prediction of myelin-specific reactivity.

In this study, we aimed to characterize the myelin reactivity and HLA class II profile in a Belgian population of MS patients and to evaluate a possible association between precursor frequency of myelin-reactive T cells and HLA haplotype. Nonetheless, we were not able to demonstrate an association between myelin responsiveness and the primordial MS-associated risk alleles of the HLA-DR15 haplotype, consisting of HLA-DRB1*15:01, HLA-DQA1*01:02 and HLA-DQB1*06:02 [15]. This is in line with findings from Bronge et al., who equally did not detect a difference in T cell response towards MOG protein between HLA-DRB1*15:01 carriers and non-carriers [29]. Similarly, we did not find an association between additional MS-associated alleles HLA-DRB3, HLA-DRB4 and HLA-DPB1*03:01 and myelin-specific reactivity.

The complex etiology of MS comprises both genetic and environmental factors [30,31]. The genetic risk for MS is strongly associated with HLA class II alleles, of which the HLA-DR15 haplotype has been studied extensively as a genetic risk factor for MS. The strong association between this haplotype and MS has been consistently reported in many studies [13]. However, the prevalence of HLA risk alleles depends on the geographical location [15]. Recently, Lysandropoulos et al. have analyzed the frequencies of HLA class I and II alleles in a population of 119 Belgian MS patients and 124 healthy controls [32]. They found the HLA-DRB1*15 allele to be more frequent in MS patients, namely 45.4% of MS patients as compared to 24.2% of healthy controls (*p* = 0.01). Moreover, the DRB1*15-DQB1*06 haplotype was more frequently observed in MS patients as compared to healthy controls (44.5% versus 23.4%, *p* = 0.01). Similar frequencies were found in this Belgian cohort, i.e., 45.0% of the patients (9/20) carried the HLA-DRB1*15:01 allele, whereas 40.0% of the patients (8/20) carried the HLA-DRB1*15:01-DQB1*06:02 haplotype. In contrast, only 10.0% (1/10) of healthy controls was a carrier of the HLA-DRB1*15:01 allele and of the HLA-DRB1*15:01-DQB1*06:02 haplotype.

In addition, we characterized the HLA class II profile of patients in more depth by analyzing the HLA-DRB3, -DRB4, -DRB5, -DQA1, -DPA1 and -DPB1 loci. The DRB3, DRB4 and DRB5 loci are presumed to act as modulators of the DRB1-associated MS risk [33]. In particular, the strong linkage disequilibrium between HLA-DRB1*15:01 and HLA-DRB5 is well-described [34] and was also confirmed in our hands, as indicated by the fact that all HLA-DRB5*01:01 carriers were HLA-DRB1*15:01 carriers. It has been hypothesized that HLA-DRB5 acts as a modifier of progression, rather than a factor for MS susceptibility, since HLA-DRB5*null patients are at higher risk for the development of secondary progressive MS [35]. Since only RR-MS patients were included in this study, long-term follow-up of our patient cohort would be of interest to evaluate the HLA-DRB5*null genotype as a risk factor for secondary progressive MS. Furthermore, we report a prevalence of 65.0% (13 out of 20 patients, of which two homozygous) for the HLA-DRB3 allele. The HLA-DRB3 gene, expressed as DR52 on the cell membrane, is in linkage as a haplotype with DRB1*03:01 and DRB1*13:03 [13]. Previously, others found 25% of Brazilian MS patients carrying the HLA-DRB3 allele [36]. Whether the difference reflects geographical variations or is due to the detection technique used needs to be elucidated. The HLA-DRB4 gene, on the other hand, expresses the DR53 antigen and is in linkage as a haplotype with DRB1*04:05 [13]. Interestingly, the HLA-DR53 haplotype was previously shown to be modestly protective for MS in a Finnish population (OR = 0.5; MS patients versus healthy controls) [33], but could not be detected in our Belgian patient cohort.

Overall, a myelin-specific IFN-γ response could be detected in 13 out of 20 patients (65.0%). Previously, Grau-Lopez et al. detected a positive proliferative reaction to a mix of seven myelin-derived peptides (MBP_13–32_, MBP_111–129_, MBP_146–170_, MBP_83–99_, MOG_1–20_, MOG_35–55_ and PLP_139–154_) in 74% of MS patients [37]. In our hands, only 6 out of 20 (33.3%) of MS patients demonstrated a positive response to these peptides. Whether the observed differences in myelin responsiveness are due to the use of a different detection technique (IFN-γ response versus proliferation test), or as a reflection of the high variability of myelin response among patient cohorts, needs further investigation. In addition, we tested T cell reactivity towards the MBP-peptides, MBP_30–44_ and MBP_131–145_. These so-called naturally-processed peptides [38] are more likely to be involved in disease pathogenesis in comparison with non-naturally-processed or cryptic epitopes [39]. However, in our hands, responses towards these naturally-processed peptides could only be demonstrated in healthy controls and not in MS patients. Hence, the exact role of these peptides in MS pathogenesis needs further investigation. In this context, additional cytokine read-out could help to further elucidate the specific nature of immune responses towards these peptides. The presence of myelin-reactive T cells could be demonstrated in both MS patients and healthy controls. The fact that myelin-reactive T cells appear to be part of the normal T cell repertoire implies that the culprit for their pathophysiological role in MS is dependent on other factors, such as frequency or functional properties. However, regarding frequency, both similar [40,41] and higher [29,42,43,44,45,46] numbers of myelin-reactive T cells have been described in MS patients compared to healthy controls. This contrast in the obtained results may be caused by differences in detection technique (proliferation versus cytokine release assays) or by different antigen sources (e.g., full-length protein- versus peptide-based assays, naturally-obtained versus recombinantly-produced protein). Indeed, high-sensitivity assays with minimal risk of increase in background signal due to contamination of the antigen source are needed in the light of the anticipated low frequency of MOG-reactive T cells. In this context, Bronge et al. have recently detected a higher number of MOG-reactive T cells in MS patients versus healthy controls, using a sensitive cytokine release assay with bead bound-MOG [29]. Moreover, they demonstrated that MOG-reactive T cells are present in almost half of MS patients. Concerning functional properties, Cao et al. recently demonstrated that myelin-reactive T cells from MS patients tend to produce primarily proinflammatory cytokines (IFN-γ, IL-17 and GM-CSF) compared to T cells from healthy subjects, producing anti-inflammatory IL-10 [47]. This may point towards functional differences between antigen-specific T cells from MS patients and healthy controls as additional driving factor in disease development.

There are a few limitations to this study. Firstly, this study was a cohort study investigating the HLA haplotype and myelin responsiveness of MS patients and healthy controls at one moment in time. Longitudinal follow-up of myelin reactivity of this cohort could be of interest to further evaluate epitope spreading. Secondly, our study was powered to detect a large effect size of HLA class II genotype on the myelin reactivity profile. Confirmation of our findings in larger cohorts would allow statistical evaluation of smaller effect sizes. Thirdly, myelin-specific responsiveness was based on secretion of one cytokine upon restimulation with myelin peptides, i.e., IFN-γ, given the established importance of this cytokine in MS pathogenesis [48]. For future research, further characterization of the myelin-specific response, including GM-CSF, IL-17 and IL-10 secretion and myelin-specific proliferation, may provide more information regarding the potential pathogenic nature of specific myelin responses.

In conclusion, the results of this study show that the majority of MS patients carries MS risk alleles and displays responsiveness to multiple myelin-derived epitopes. However, no association between HLA haplotype and myelin reactivity could be demonstrated in our hands. These findings are of importance for the development of new antigen-specific therapeutic interventions, aiming to restore tolerance towards myelin-derived proteins. While these antigen-specific strategies are gaining interest as a potential treatment option for MS, the use of different myelin-derived peptides, of which some are HLA-restricted [49,50], may not be sufficient to fully address the wide variation in myelin-specific responses and HLA profiles in a heterogeneous group of MS patients. Therefore, next-generation antigen-specific treatment approaches may benefit from the use of other strategies for antigen administration, including nucleic acids encoding myelin proteins, either by direct vaccination or by administration of nucleic acid-transfected antigen-presenting cells. In doing so, induction of tolerance may be achieved by targeting myelin-reactive T cells in a multi-epitope and HLA-unrestricted manner.

## Figures and Tables

**Table 1:** Clinical details of the study subjects included into the study.

MS Patients							Healthy Controls		
Code	Gender	Age	Disease Duration (Years)	EDSS Score	Current Treatment (Treatment Duration in Years)	Active MS	Code	Gender	Age
**100**	male	62	25	6.5	glatiramer acetate (16)	no	**V1**	male	23
**101**	female	69	32	6.0	-	no	**V2**	male	46
**102**	female	39	12	1.5	dimethyl fumarate (0.5)	no	**V3**	female	39
**103**	female	49	14	3.0	-	yes	**V4**	male	27
**104**	female	52	4	2.5	glatiramer acetate (0.5)	no	**V5**	female	50
**105**	male	31	10	1.0	interferon-β (9)	no	**V6**	female	56
**106**	female	66	23	2.0	glatiramer acetate (24)	no	**V7**	female	22
**107**	female	27	3	2.0	-	yes	**V8**	female	34
**108**	female	24	5	0	dimethyl fumarate (5)	no	**V9**	female	64
**109**	male	62	19	2.5	interferon-β (19)	no	**V10**	male	64
**110**	male	53	16	3.0	interferon-β (8)	no			
**112**	male	24	2	2.5	dimethyl fumarate (2)	no			
**113**	female	27	11	2.0	dimethyl fumarate (3)	no			
**114**	male	46	1	2.0	-	no			
**115**	male	50	1	1.0	dimethyl fumarate (1)	no			
**116**	female	30	5	1.0	dimethyl fumarate (3)	no			
**117**	male	53	16	3.5	-	no			
**118**	female	28	1	0	-	no			
**119**	female	41	1	2.0	dimethyl fumarate (1)	no			
**121**	female	42	9	2.0	glatiramer acetate (9)	no			
**Median** **(range)**		44 (24–69)	9.5 (1–32)	2.0 (0–6.5)					42.5 (22–64)

Active multiple sclerosis (MS) was defined as the occurrence of at least one clinical relapse in the previous year and/or at least one new or enlarging T2 lesion on magnetic resonance imaging (MRI) in the previous year. Abbreviations used: RR-MS, relapsing-remitting multiple sclerosis; EDSS, Expanded Disability Status Scale.

**Table 2:** Myelin reactivity of study subjects.

	Subject Code	MOG_1–20_	MOG_35–55_	MOG_64–86_	MOG_74–96_	MBP_13–32_	MBP_30–44_	MBP_34–56_	MBP_111–129_	MBP_131–145_	MBP_146–170_	PLP_139–154_
MS patients	100	-	-	-	**x**	-	-	-	-	-	-	-
	101	-	-	**x**	**x**	-	-	-	-	-	-	-
	102	-	-	-	-	**x**	-	**x**	-	-	-	-
	103	-	-	**x**	-	-	-	-	-	-	-	-
	104	-	-	-	-	-	-	-	-	-	-	-
	105	-	-	-	-	-	-	-	-	-	-	-
	106	-	-	-	-	-	-	**x**	-	-	-	-
	107	**x**	**x**	**x**	-	-	-	-	-	-	-	-
	108	-	**x**	-	**x**	-	-	**x**	-	-	-	-
	109	-	-	**x**	**x**	-	-	-	-	-	**x**	-
	110	-	-	**x**	**x**	-	-	-	-	-	-	-
	112	-	-	-	-	-	-	-	-	-	-	-
	113	-	-	-	-	-	-	-	-	-	-	-
	114	-	-	**x**	-	-	-	-	-	-	-	-
	115	-	-	-	-	-	-	-	-	-	-	-
	116	-	-	-	-	-	-	-	-	-	-	-
	117	**x**	-	-	-	-	-	-	-	-	-	-
	118	-	-	-	-	-	-	-	-	-	-	-
	119	**x**	**x**	-	**x**	**x**	-	**x**	-	-	**x**	**x**
	121	-	-	**x**	-	-	-	-	-	-	-	-
	*Total n° of responses*	*3*	*3*	*7*	*6*	*2*	*0*	*4*	*0*	*0*	*2*	*1*
Healthy controls	V1	-	**x**	**x**	-	-	-	-	-	-	-	-
	V2	-	-	-	-	-	-	-	-	-	-	**x**
	V3	-	-	-	-	-	-	-	-	-	-	-
	V4	**x**	-	**x**	**x**	-	-	-	-	-	-	-
	V5	-	-	-	-	-	-	-	-	-	-	-
	V6	-	-	-	-	-	**x**	-	-	-	-	-
	V7	-	**x**	**x**	**x**	**x**	**x**	**x**	**x**	**x**	**x**	**x**
	V8	-	-	-	-	-	-	-	-	-	-	-
	V9	-	-	-	-	-	-	-	-	-	-	-
	V10	-	-	**x**	-	-	-	-	-	-	-	-
	*Total n° of responses*	*1*	*2*	*4*	*2*	*1*	*2*	*1*	*1*	*1*	*1*	*2*

For 11 individual myelin peptides, presence of PBMC reactivity is indicated in grey. A peptide-specific response was defined as present when >50% of the peptide-restimulated wells were positive. A well was defined as positive when the number of spot-forming cells in the well was higher than the [mean spot forming cells + 3 standard deviations (S.D.)] of the negative control wells. A subject displaying at least one peptide-specific response was defined as a responder. Abbreviations used: PBMC, peripheral blood mononuclear cells; MOG, myelin oligodendrocyte glycoprotein; MBP, myelin basic protein; PLP, proteolipid protein.

**Table 3:** Human leukocyte antigen (HLA) class II genotyping of study subjects.

	Code	DRB1		DQA1		DQB1		DRB3		DRB4	DRB5	DPA1		DPB1	
MS patients	100	*03:01	*01:01	*05:01	*01:01	*02:01	*05:01	*02:02	-	-	-	*01:03	*01:03	*04:01	*02:01
	101	*13:02	*08:03	*01:02	*06:01	*06:09	*03:01	*03:01	-	-	-	*01:03	-	*02:01	*16:01
	102	*03:01	*01:02	*05:01	*01:01	*02:01	*05:01	*01:01	-	-	-	*02:01	*01:03	*17:01	*02:01
	103	*15:01	*03:01	*01:02	*05:01	*06:03	*02:01	*01:01	-	-	*01:01	*02:01	*01:03	*09:01	*04:01
	104	*11:01	*15:01	*05:09	*01:02	*03:01	*06:02	*02:02	-	-	*01:01	*01:03	*02:06	*03:01	*05:01
	105	*15:01	*01:01	*01:02	*01:01	*06:02	*05:01	-	-	-	*01:01	*01:03	*02:01	*04:02	*14:01
	106	*15:01	-	*01:02	-	*06:02	-	-	-	-	*01:01	*01:03	-	*04:01	-
	107	*15:01	*01:02	*01:02	*01:01	*05:01	*06:02	-	-	-	*01:01	*01:03	*01:03	*04:02	*02:01
	108	*15:01	*08:XX	*01:02	*04:01	*06:02	*04:02	-	-	-	*01:01	*01:03	-	*04:01	-
	109	*11:01	*01:01	*05:05	*01:01	*03:01	*05:01	*02:02	-	-	-	*01:03	*01:03	*04:02	*04:01
	110	*12:01	*01:01	*05:05	*01:01	*03:01	*05:01	*02:02	-	-	-	*01:03	*01:03	*02:01	*03:01
	112	*11:02	*10:01	*05:05	*01:05	*03:19	*05:01	*02:02	-	-	-	*01:03	*02:01	*04:01	*30:01
	113	*07:01	*15:01	*02:01	*01:02	*02:02	*06:02	-	-	*01:01	*01:01	*01:03	*02:01	*04:01	*11:01
	114	*13:02	*03:01	*01:02	*05:01	*06:04	*02:01	*03:01	*01:01	-	-	*02:01	*01:03	*01:01	*03:01
	115	*03:01	*15:01	*05:01	*01:02	*02:01	*06:02	*01:01	-	-	*01:01	*02:01	*01:03	*01:01	*04:01
	116	*15:01	*08:01	*01:02	*04:01	*06:02	*04:02	-	-	-	*01:01	*01:03	*01:03	*04:02	-
	117	*04:01	*03:01	*03:01	*05:01	*03:02	*02:01	*01:01	-	*01:03	-	*01:03	*01:03	*04:01	-
	118	*11:04	*10:01	*05:05	*01:05	*03:01	*05:01	*02:02	-	-	-	*01:03	*01:03	*02:01	*04:02
	119	*07:01	*01:01	*02:01	*01:02	*03:03	*05:04	-	-	*01:03:01:02N	-	*01:03	*01:03	*02:01	*04:01
	121	*03:01	*13:02	*05:01	*01:02	*02:01	*06:04	*01:01	*03:01	-	-	*01:03	*02:02	*04:01	*01:01
Healthy controls	V1	*07:01	*03:01	*02:01	*05:01	*02:02	*02:01	*01:01	-	*01:03	-	*02:01	*01:03	-	-
	V2	*07:01	*12:01	*02:01	*05:05	*03:03	*03:01	*02:02	-	*01:03:01:02N	-	*01:03	*01:03	-	-
	V3	*14:54	*07:01	*01:04	*02:01	*05:03	*02:02	*02:02	-	*01:01	-	*01:03	*01:03	-	-
	V4	*03:01	*01:01	*05:01	*01:01	*02:01	*05:01	*02:02	-	-	-	*01:03	*01:03	-	-
	V5	*07:01	*03:01	*02:01	*05:01	*02:02	*02:01	*01:01	-	*01:03:01:02N	-	*02:01	*01:03	*11:01	*04:02
	V6	*16:01	*04:01	*01:02	*03:03	*05:02	*03:02	-	-	*01:03	*02:02	*02:01	*01:03	*01:01	*04:01
	V7	*13:01	-	*01:03	-	*06:03	-	*02:02	*01:01	-	-	*01:03	*02:02	*04:02	*05:01
	V8	*07:01	*03:01	*02:01	*05:01	*02:02	*02:01	*01:01	-	*01:03	-	*01:03	-	*04:01	-
	V9	*03:01	*15:01	*05:01	*01:02	*02:01	*06:02	*01:01	-	-	*01:01	*02:01	*01:03	-	-
	V10	*13:05	*14:54	*05:05	*01:04	*03:01	*05:03	*02:02	*02:02	-	-	*01:03	*02:01	-	-

Patients with the HLA-DR15 haplotype (combination of the risk alleles HLA-DRB1*15:01, HLA-DQA1*01:02, HLA-DQB1*06:02) are highlighted in orange. HLA-DRB1*08:XX is a new variant, similar to DRB1*08:01, but with a point mutation in exon 3 (valine-methionin substitution). In the table, “-” refers to negative; an allele could not be detected.

**Table 4:** Overview of the myelin peptide-specific T cell responses among carriers and non-carriers of MS-associated HLA susceptibility alleles.

		HLA-DR15		HLA-DRB1*15:01		HLA-DQA1*01:02		HLA-DQB1*06:02	
		+ (9/30)	- (21/30)	*+* *(10/30)*	*-* *(20/30)*	*+* *(15/30)*	*-* *(15/30)*	*+* *(9/30)*	*-* *(21/30)*
**MOG**									
All MOG	Response	2	13	3	12	6	9	2	13
MOG_1–20_	Response	1	3	1	3	2	2	1	3
	Frequency	78.5 ± 185.8	49.2 ± 50.4	73.0 ± 176.0	50.5 ± 51.3	68.3 ± 147.0	47.7 ± 43.7	78.5 ± 185.8	49.2 ± 50.4
MOG_35–55_	Response	2	2	2	3	3	2	2	2
	Frequency	88.9 ± 155.1	45.5 ± 49.0	81.5 ± 148.1	47.0 ± 49.8	68.0 ± 121.1	49.0 ± 56.4	88.9 ± 155.1	45.5 ± 49.0
MOG_64–86_	Response	1	10	2	9	5	6	1	10
	Frequency	39.5 ± 60.4	91.5 ± 108.8	50.6 ± 66.8	68.2 ± 93.3	47.7 ± 55.2	104.1 ± 124.3	39.5 ± 60.4	91.5 ± 108.8
MOG_74–96_	Response	1	7	1	7	3	5	1	7
	Frequency	40.7 ± 52.7	45.8 ± 34.7	43.3 ± 50.3	49.3 ± 41.5	45.7 ± 45.6	42.9 ± 35.0	40.7 ± 52.7	45.8 ± 34.7
**MBP**									
All MBP	Response	2	5	2	5	4	3	2	5
MBP_13–32_	Response	0	3	0	3	1	2	0	3
	Frequency	20.1 ± 18.4	39.8 ± 32.1	23.4 ± 20.3	30.8 ± 20.4	25.9 ± 21.0	41.8 ± 35.6	20.1 ± 18.4	39.8 ± 32.1
MBP_30–44_	Response	0	2	0	2	1	1	0	2
	Frequency	21.1 ± 19.1	27.2 ± 19.3	20.8 ± 18.1	26.0 ±18.0	19.5 ± 15.1	31.2 ± 21.4	21.1 ± 19.1	27.2 ± 19.3
MBP_34–56_	Response	2	3	2	3	3	2	2	3
	Frequency	40.8 ± 45.2	30.6 ± 22.7	40.9 ± 42.6	32.5 ± 23.5	41.3 ± 39.8	26.1 ± 15.5	40.8 ± 45.2	30.6 ± 22.7
MBP_111–129_	Response	0	1	0	1	0	1	0	1
	Frequency	22.0 ± 15.9	24.4 ± 15.3	21.8 ±15.0	23.3 ± 15.2	19.4 ± 12.8	28.0 ± 16.6	22.0 ± 15.9	24.4 ± 15.3
MBP_131–145_	Response	0	1	0	1	0	1	0	1
	Frequency	27.4 ± 23.8	25.0 ± 17.2	26.1 ± 22.7	25.1 ±17.6	23.4 ± 19.0	28.0 ± 19.4	27.4 ± 23.8	25.0 ± 17.2
MBP_146–170_	Response	0	3	0	3	1	2	0	3
	Frequency	22.9 ± 21.6	28.7 ± 23.4	22.0 ± 20.6	30.4 ± 24.1	25.5 ± 28.0	28.3 ± 16.7	22.9 ± 21.6	28.7 ± 23.4
**PLP**									
PLP_139–154_	Response	0	3	0	3	1	2	0	3
	Frequency	26.6 ± 14.6	30.6 ± 20.6	25.6 ± 14.1	33.3 ± 20.7	29.1 ± 21.7	29.8 ± 16.3	26.6 ± 14.6	30.6 ± 20.6
**All peptides**									
Response to at least one peptide		3	16	4	15	9	10	3	16

The number of study subjects demonstrating a peptide-specific T cell response is indicated per MS susceptibility HLA allele. A peptide-specific response was defined as present when >50% of the peptide-restimulated wells were positive. A well was defined as positive when the number of spot-forming cells in the well was higher than the [mean spot forming cells + 3 standard deviations (S.D.)] of the negative control wells. The frequency of peptide-reactive T cells is expressed as mean ± standard deviation. No statistically significant difference in the presence of a peptide-specific T cell response or in the frequency of peptide-reactive T cells could be demonstrated between carriers and non-carriers of a particular susceptibility HLA allele, using Fisher’s exact test and a two-way ANOVA, respectively. Abbreviations used: MOG, myelin oligodendrocyte glycoprotein; MBP, myelin basic protein; PLP, proteolipid protein; PBMC, peripheral blood mononuclear cells; HLA, human leukocyte antigen.
